# Gut mobilization improves behavioral symptoms and modulates urinary p‐cresol in chronically constipated autistic children: A prospective study

**DOI:** 10.1002/aur.2639

**Published:** 2021-11-23

**Authors:** Laura Turriziani, Arianna Ricciardello, Francesca Cucinotta, Fabiana Bellomo, Giada Turturo, Maria Boncoddo, Silvestro Mirabelli, Maria Luisa Scattoni, Maddalena Rossi, Antonio M. Persico

**Affiliations:** ^1^ Interdepartmental Program "Autism 0‐90" "G. Martino" University Hospital of Messina Messina Italy; ^2^ IRCCS Centro Neurolesi "Bonino‐Pulejo" Messina Italy; ^3^ Research Coordination and Support Service, Istituto Superiore di Sanità Rome Italy; ^4^ Department of Life Sciences & BIOGEST‐SITEIA University of Modena and Reggio Emilia Modena Italy; ^5^ Child & Adolescent Neuropsychiatry Program, Modena University Hospital, & Department of Biomedical, Metabolic and Neural Sciences University of Modena and Reggio Emilia Modena Italy

**Keywords:** 4‐cresol, anxiety, autism, autism spectrum disorder, biomarkers, constipation, microbiota

## Abstract

Chronic constipation is common among children with ASD and is associated with more severe hyperactivity, anxiety, irritability, and repetitive behaviors. Young autistic children with chronic constipation display higher urinary, and foecal concentrations of p‐cresol, an aromatic compound produced by gut bacteria, known to negatively affect brain function. Acute p‐cresol administration to BTBR mice enhances anxiety, hyperactivity and stereotypic behaviors, while blunting social interaction. This study was undertaken to prospectively assess the behavioral effects of gut mobilization in young autistic children with chronic constipation, and to verify their possible correlation with urinary p‐cresol. To this aim, 21 chronically constipated autistic children 2–8 years old were evaluated before (T0), 1 month (T1), and 6 months (T2) after intestinal mobilization, recording Bristol stool scale scores, urinary p‐cresol concentrations, and behavioral scores for social interaction deficits, stereotypic behaviors, anxiety, and hyperactivity. Gut mobilization yielded a progressive and highly significant decrease in all behavioral symptoms over the 6‐month study period. Urinary p‐cresol levels displayed variable trends not significantly correlated with changes in behavioral parameters, mainly increasing at T1 and decreasing at T2. These results support gut mobilization as a simple strategy to ameliorate ASD symptoms, as well as comorbid anxiety and hyperactivity, in chronically constipated children. Variation in p‐cresol absorption seemingly provides limited contributions, if any, to these behavioral changes. Further research will be needed to address the relative role of reduced abdominal discomfort following mobilization, as compared to specific modifications in microbiome composition and in gut bacteria‐derived neuroactive compounds.

## Lay Summary

Many autistic children suffer from chronic constipation. Gut mobilization in 21 chronically constipated autistic children followed prospectively for 6 months, consistently reduced hyperactivity, anxiety, sociocommunication deficits, restricted interests, and stereotypic behaviors. Changes in urinary p‐cresol, a gut bacteria‐derived neuroactive compound able to negatively affect brain function in rodent models, was not correlated with behavioral parameters, except for a marginal association with changes in anxiety. Gut mobilization significantly improves behavioral symptoms in chronically constipated autistic children, through multiple mechanisms possibly including, but not limited to, reduction in p‐cresol absorption.

## INTRODUCTION

Autism Spectrum Disorder (ASD) is a complex and heterogenous neurodevelopmental disorder, beginning in early childhood and with a lifelong course in most cases. It is characterized by impairment in social interaction and communication, as well as at least two among repetitive behaviors, insistence on sameness, restricted interests, and abnormal sensory processing (American Psychiatric Association, 2013). Its incidence has dramatically risen during the last few decades, reaching the prevalence of 1/54 children and 1/45 adults in the United States (Dietz et al., 2020; Maenner et al., 2020), 1/87 children in Italy and 1/102 adults in England (Brugha et al., 2011; Narzisi et al., 2018). Indeed, genetics has been shown to play a major role in ASD pathogenesis, but many cases remain unexplained even after the most advanced genetic testing. In fact, the etiology of autism often implicates multiple causes, genetic and/or environmental, determining the autism phenotype through complex interactions whose precise underlying mechanisms remain poorly understood (Bölte et al., 2019; Liu et al., 2021).

ASD does not affect neurodevelopment exclusively in the central nervous system (CNS). Associated symptoms frequently include gastrointestinal (GI) disorders, like chronic constipation, diarrhea, alternating stool, gastroesophageal reflux, frequent vomiting, and feeding problems, often producing abdominal pain (Lefter et al., 2019; Mayer et al., 2014). Chronic constipation has been generally reported as the most frequent GI disorder, present either alone or in combination with other GI symptoms in up to 80% of autistic children (Buie et al., 2010; Chakraborty et al., 2021; Gorrindo et al., 2012; Ferguson et al., 2019; Lefter et al., 2019), reaching the impressive OR of 3.86 (CI: 2.23–6.71) in ASD compared to neurotypical children (McElhanon et al., 2014).

GI symptoms may well stem from abnormal functioning of the enteric nervous system (ENS), which in humans comprises 200–600 million neurons entirely contained within the gut wall (Furness et al., 2014). As a paradigmatic example, ASD‐associated human mutations in the *CHD8* locus reduce intestinal motility by blunting the population of ENS neurons present in the zebrafish gut wall (Bernier et al., 2014). In addition, food selectivity, limited fluid intake and more rarely sensory issues with passing stool may also contribute to constipation (Harris et al., 2021). However, two additional lines of investigation highlight a more complex scenario, based on the bidirectional communication between the gut and the brain. On the one hand, autistic children often carry an intestinal dysbiosis, which can exacerbate both CNS and GI malfunctioning in several ways (Wang et al., 2011). For example, a skewed ASD‐associated gut microbiota can boost the production of inflammatory cytokines, increasing intestinal permeability (Fattorusso et al., 2019), and can negatively influence mRNA splicing in the CNS, yielding ASD‐like behaviors in rodent models (Sharon et al., 2019). On the other hand, urinary and foecal metabolomic studies performed in recent years have identified a set of small molecules significantly elevated in autistic children compared to matched controls, comprising metabolites produced by the gut microbiota and not of human origin (Gevi et al., 2016; Mussap et al., 2020). Interestingly, some of these metabolites, like p‐cresol, its conjugated form p‐cresyl‐sulfate, and propionic acid, are neuroactive (Bermudez‐Martin et al., 2021; MacFabe, 2015; Pascucci et al., 2020; Persico & Napolioni, 2013).

In autistic children, the presence of gastrointestinal disorders, especially constipation, has been consistently associated with more severe behavioral symptoms (Chakraborty et al., 2021; Ferguson et al., 2019; Fulceri et al., 2016; Gorrindo et al., 2012; Mazurek et al., 2013; Peters et al., 2014; Prosperi et al., 2019; Restrepo et al., 2020). Conversely, gut mobilization obtained using prebiotics and/or probiotics may reduce irritability and improve some behavioral parameters (Grimaldi et al., 2018; Inoue et al., 2019; Sanctuary et al., 2019), while long‐term behavioral improvements have been reported after microbiota transfer therapy (Kang et al., 2017; Kang et al., 2019). Hence, the presence of gut dysbiosis able to influence CNS function in multiple ways through the production of neuroactive metabolites suggests that correlations between GI issues and behavior in ASD may also stem from specific modulation of neurodevelopment and brain function, above and beyond the non‐specific relief from abdominal discomfort produced by therapeutic interventions.

P‐cresol (4‐methylphenol) can be detected in bodily fluids, like the urines, of most individuals as the result of either environmental exposure and absorption through the skin, GI, and respiratory systems, or bacterial fermentation of the aromatic amino acids phenylalanine and tyrosine through the action of p‐hydroxyphenylacetate decarboxylase and/or tyrosine lyase (Persico & Napolioni, 2013; Saito et al., 2018). Intestinal bacteria producing p‐cresol include several species mainly belonging to *Coriobacteriaceae* and *Clostridium* clusters XI and XIVa, with *Blautia hydrogenotrophica*, *Clostridium difficile*, *Olsenella uli*, and *Romboutsia lituseburensis* identified as particularly strong producers (Saito et al., 2018). Some of these species, especially *Clostridium difficile* in cluster IX, have been found over‐represented in the microbiome of autistic individuals (Kelly et al., 2017; Sharon et al., 2019). Targeted studies have detected significantly elevated concentrations of p‐cresol in the urines of young autistic children until age 8 both in Italy and in France (Altieri et al., 2011; Gabriele et al., 2014). In these initial studies, urinary p‐cresol showed some association with autism severity or with the intensity of abnormal behaviors (Altieri et al., 2011; Gabriele et al., 2014). Subsequently, several unbiased urinary or foecal metabolomic studies consistently detected p‐cresol among the metabolites significantly increased in ASD children compared to matched controls (De Angelis et al., 2013; Gevi et al., 2016; Kang et al., 2018; Kang et al., 2020; Mussap et al., 2020). Acute p‐cresol administration to BTBR mice, a widely utilized rodent model of ASD, has been found to exacerbate abnormal behaviors relevant to ASD, namely anxiety in the elevated plus maze and hyperactivity in the open field at a lower dose (1 mg/kg i.v.), stereotypic behaviors in the open field and loss of social preference in the three‐chamber Social Interaction Test at a higher dose (10 mg/kg i.v.) (Pascucci et al., 2020). These same animals displayed enhanced dopaminergic turnover in amygdala as well as in dorsal and ventral striatum, a regional distribution and dose‐dependency tightly coherent with the observed behavioral abnormalities (Pascucci et al., 2020). Interestingly, p‐cresol has long been known to inhibit dopamine‐*β*‐hydroxylase (Goodhart et al., 1987) and through this mechanism it could foster neurochemical imbalances in line with the proposed “dopaminergic hypothesis” of ASD (DiCarlo et al., 2019; Pavăl, 2017). Very recently, these results have been partly replicated and extended in C57BL/6J male mice using an oral 4‐week administration paradigm: social behavior deficits seemingly stem from cresol‐induced changes in the composition of the gut microbiota, can be transferred from p‐cresol‐treated mice to control mice by fecal microbiota transplantation, and are primarily associated with changes in dopaminergic neurotransmission (Bermudez‐Martin et al., 2021).

Several years ago, we reported that urinary p‐cresol was elevated specifically in autistic children with chronic constipation, supporting the hypothesis that increased intestinal transit time may foster greater production and absorption of this neuroactive compound (Gabriele et al., 2016). The multiple lines of evidence summarized above, today suggest that chronic constipation, by promoting proteolytic metabolism, altering the composition of the gut microbiota, and causing greater absorption of p‐cresol, may ultimately produce larger neurochemical effects on dopaminergic turnover in amygdala, n. accumbens and dorsal striatum, worsening anxiety, hyperactivity, stereotypic behaviors, and socio‐communication deficits in children with ASD due to different genetic or gene–environment interaction mechanisms. The present study was undertaken to test this hypothesis, by prospectively measuring behavioral symptoms, urinary p‐cresol concentrations, and stool consistency in chronically constipated ASD children 2–8 years old, before (T0), 1 month (T1), and 6 months (T2) after intestinal mobilization.

## METHODS

### Patient sample

A sample of 25 children was initially recruited at the Interdepartmental Program “Autism 0–90” of the “G. Martino” University Hospital in Messina (Italy), based on: (1) fulfilling DSM‐5 diagnostic criteria for Autism Spectrum Disorder, (2) confirming the diagnosis using the Autism Diagnostic Observation Schedule, 2nd ed. (ADOS‐2) (Lord et al., 2012), (3) age 2–8 years old, in accordance with prior studies indicating that significant increases in urinary p‐cresol concentrations are detected in ASD up until age 8 (Altieri et al., 2011; Gabriele et al., 2014), and (4) chronic constipation, namely unsatisfactory defecation characterized by difficult and infrequent passage of lumpy and hard stools during at least the previous 3 months, as reported by parents based on the Bristol Stool Scale (Lewis & Heaton, 1997). This Scale classifies human feces based on shape and consistency, into seven categories, ranging from extreme constipation (type 1: “separate hard lumps”) to severe diarrhoea (type 7: “liquid consistency with no solid pieces”). Four subjects assessed at T0 were lost at T1 and four additional children assessed at T0 and T1 were lost at T2 due to the COVID‐19 pandemic. Hence, data reported in this study refer to a sample size of 21 children at T0 and T1, and 17 children at T2. At the time of recruitment, all 21 participants had a score of 1 or 2 at the Bristol Stool Scale (Lewis & Heaton, 1997), except for one child who had a score of 3, with a documented lifelong history of constipation. All parents gave written informed consent for their children, using the consent form approved by the Ethical Committee of the University of Messina (Messina, Italy). This open trial has been registered in https://clinicaltrials.gov/ with NCT no. 05025553.

### Study design

At the time of recruitment, Intellectual Quotient (IQ) or Developmental Quotient (DQ) were determined using the Leiter International Performance Scale‐Third Edition (Roid et al., 2013) or the Griffith Mental Development Scales (Luiz et al., 2006), respectively. Autistic behaviors were assessed using the ADOS‐2 (Lord et al., 2012), and the Children Autism Rating Scales (CARS) (Schopler et al., 1986). Adaptive functioning was assessed using the Vineland Adaptive Behavior Scales‐Second Edition (Sparrow et al., 2005).

Following this initial assessment, all children underwent gut mobilization using a standard protocol with oral administration of polyethylene glycol (PEG) at the dose of 6.9 g/day once a day for 6 months. Children were observed at baseline (T0), 1 month (T1), and 6 months (T2) after gut mobilization. At each time point, urines were collected to measure p‐cresol, stool quality was assessed by parental report using the Bristol Stool Scale (Lewis & Heaton, 1997), and parents filled in the Repetitive Behavior Scale‐Revised (RBS‐R) (Bodfish et al., 2000), Conners' Parent Rating Scale—Revised (CPRS‐R) (Conners et al., 1998), and Social Responsiveness Scale (SRS) (Constantino & Gruber, 2005), whereas ASD severity was measured using the CARS (Schopler et al., 1986), administered by the same psychologist at all three time points for each subject. During this 6‐month prospective study, both pharmacological treatments and behavioral interventions had to remain absolutely constant, to ensure that gut mobilization was the only independent variable able to produce behavioral changes. Use of antibiotics due to infections during the study was not a cause of exclusion, but was recorded by parental report.

### Urine collection and p‐cresol measurement by HPLC

First‐morning urines were collected at home by parents at T0, T1, and T2 using sterile containers and were brought to the clinical center the same morning in wet ice. Skin adhesive pediatric urine collection bags were utilized for younger children not yet toiled trained, following instructions provided by the manufacturer. Urine samples were then frozen, shipped in dry ice, and stored at −80°C until analysis. Urinary concentrations of total p‐cresol, encompassing on average 95% p‐cresylsulfate, 3%–4% p‐cresylglucuronide and 0.5%–1% of unconjugated free p‐cresol (Persico & Napolioni, 2013), were measured by High Performance Liquid Chromatography (HPLC) with fluorescence detector and quantified after acid hydrolysis, as previously described (Altieri et al., 2011; Gabriele et al., 2014). The correlation coefficient of the calibration straight lines was always >0.999. The limit of detection, calculated as three times the height of baseline long‐term noise, was 20 ng/ml, and the limit of quantification was 70 ng/ml. Since creatinine excretion may be abnormally reduced in ASD children, data were normalized by urinary specific gravity, as in previous studies (Altieri et al., 2011; Gabriele et al., 2014).

### Statistical analyses

Kendall's *τ* statistics was used for correlation analysis. The three time points were contrasted applying one‐way ANOVAs for repeated measures followed by pairwise contrasts, using the GLM procedure available in SPSS (ver. 27). When the assumption of sfericity was not satisfied, based on Mauchly's W statistics, either Greenhouse–Geisser or Huynh–Feldt corrections were applied, depending on whether *ε* was < or ≥0.75, respectively. One‐way ANOVAs for repeated measures involved only the 17 patients with complete data at the three time points, whereas regression analysis was performed on the entire sample following Sqrt data transformation to reduce skewness and better approximate a normal distribution. Power (1 − *β*) and effect size (*η* ^2^) calculations were performed as part of the above‐mentioned procedures, assuming *α* = 0.05 and *β* = 0.20. Data are presented as mean ± SEM or median and range. Statistical significance is set at *p* < 0.05, applying Bonferroni correction for multiple testing to *F* statistics for each behavioral test separately, as several variables present in different tests are not independent. Sidak correction was applied to pairwise contrasts.

## RESULTS

The demographic and clinical characteristics of the patient sample are summarized in Table 1. All were Caucasians of Italian ethnicity, with mean (±SEM) age of 4.6 ± 1.7 years, and an M:F ratio of 6:1. Autism severity was defined by two experienced clinicians at level 1 (“requiring support”) for 5 patients, at level 2 (“requiring substantial support”) for 12 patients and at level 3 (“requiring very substantial support”) for 4 patients (Table 1).

**TABLE 1:** Demographic and clinical characteristics of the sample

	*N*	Mean ± SEM	Range
Age (years)	*N* = 21	4.55 ± 1.7	2.5–8
		**Percent**	
Gender			
Male	18	85.7%	
Female	3	14.3%	
M/F ratio	6/1		
ASD severity			
Level 1	5	23.8%	
Level 2	12	57.1%	
Level 3	4	19.1%	
I.Q. or D.Q.			
>70	7	33.3%	
≤70	14	66.7%	

	Median	Range
VABS‐II score	71	49–107
CARS scores (range 1–4)		
(1) Social relationship	2.9	2.0–4.0
(2) Imitation	2.8	1.5–4.0
(3) Emotional response	2.5	1.0–3.5
(4) Use of body	2.6	1.0–4.0
(5) Use of objects	2.5	1.0–4.0
(6) Mental and behavioral flexibility	2.3	1.0–3.5
(7) Visual response	2.4	1.0–3.5
(8) Hearing response	2.1	1.0–3.0
(9) Use of senses	2.0	1.0–3.5
(10) Fear and anxiety	1.9	1.0–4.0
(11) Verbal communication	3.0	1.5–4.0
(12) Nonverbal communication	2.6	1.5–4.0
(13) Activity level	2.5	1.0–4.0
(14) Cognitive level	2.6	1.5–4.0
(15) General impression	3.0	1.5–4.0
Total score	37.8	27.5–55.0

**FIGURE 1:**
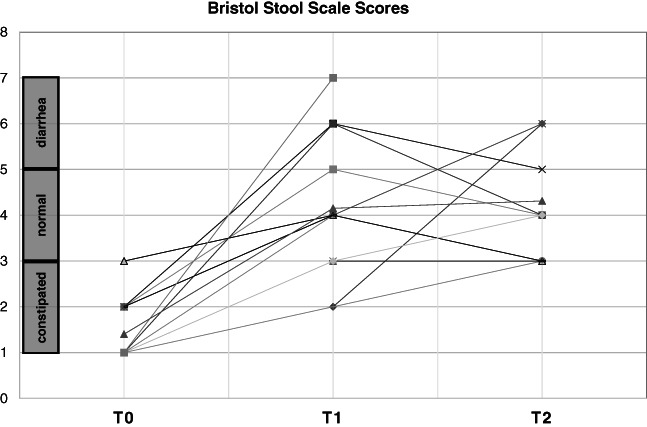
Bristol stool scale scores at baseline (T0), and after 1 (T1) and 6 months (T2) of gut mobilization

Gut mobilization using PEG consistently improved constipation in all 21 ASD children, raising Bristol stool scale scores either into the normal or diarrhea range in 19/21 (90.5%) children at 1 month and in 17/17 (100%) children at 6 months (one‐way ANOVA for repeated measures: *F* = 46.06, 2 d.f., *p* = 3.81 × 10^−10^) (Figure 1). Diarrhea was the only side effect recorded in three children at 1 month and in two children at 6 months. This side effect was mild and easily manageable according to parents, causing no patient to drop out from the study (all drop‐outs were due to the COVID‐19 pandemics).

**FIGURE 2:**
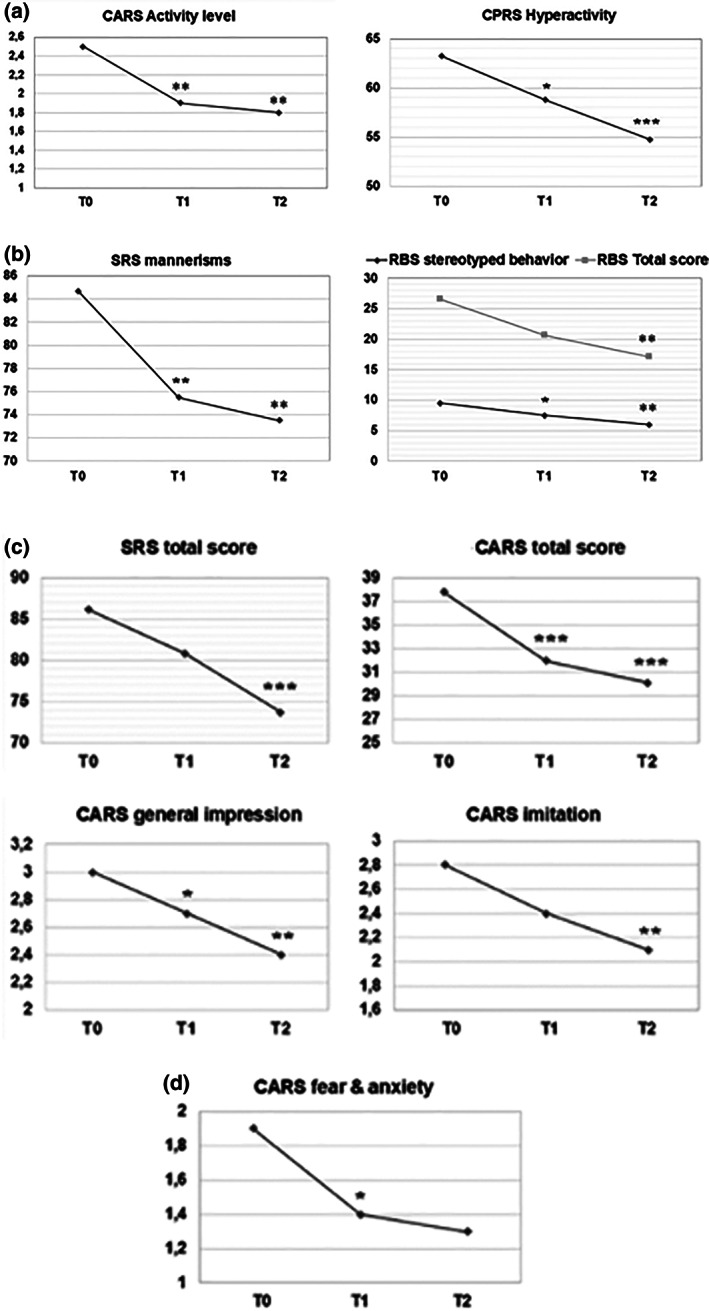
Variation in behavioral parameters from baseline (T0), after 1 (T1), and 6 months (T2) of gut mobilization: (a) CARS and CPRS subitems for hyperactivity; (b) SRS and RBS subitems for stereotypic behaviors, mannerisms and restricted interests; (c) CARS and SRS subitems for social interaction deficits (total scores, general impression, imitation); (d) CARS subitem for fears and anxiety (see Table 2 for more details). *p < 0.05; **p < 0.01; ***p < 0.001

Behavioral assessments were focused on hyperactivity, anxiety, sociocommunication deficits, and stereotypies, based on our prior rodent study (Pascucci et al., 2020). All four behavioral domains consistently improved during the 6 months following gut mobilization, albeit to a different extent (Figure 2a–d, Table 2). A significant decrease in hyperactivity was recorded, both using the CARS (item 13 “Activity level”: −25.3% at T1 and −26.5% at T2; *F* = 9.735, 2 df, *p* = 5.0 × 10^−4^; T0 > T1 = T2, *p* < 0.01) and the CPRS (“Hyperactivity”: −7.7% at T1 and −12.7% at T2; *F* = 9.515, 2 df, *p* = 0.001; T0 > T1 = T2, *p* < 0.05–0.01) (Figure 2a, Table 2). A pronounced decrease was also recorded in RBS “stereotyped behavior” (−18.4% at T1 and −39.3% at T2; *F* = 10.38, 2 df, *p* = 3.4 × 10^−4^; T0 > T1 = T2, *p* < 0.05–0.01) and RBS “total score” (−18.3% at T1 and −37.7% at T2; *F* = 11.536, 2 df, *p* = 1.7 × 10^−4^; T0 = T1 *p* = 0.053 n.s., T0 > T2 *p* < 0.01, T1 = T2 *p* = 0.116 n.s.), as well as in SRS “mannerisms” (−13.4% at T1 and −15.9% at T2; *F* = 10.762, 2 df, *p* = 2.7 × 10^−4^; T0 > T1 = T2, *p* < 0.01) (Figure 2b, Table 2). Social interaction deficits progressively improved going from T0 to T1, down to T2, as documented by SRS “total score” (−7.1% and −14.1%, *F* = 11.549, 2 df, *p* = 1.7 × 10^−4^; T0 = T1, *p* = 0.10 n.s., T0 > T2 *p* < 0.001, T1 = T2 *p* = 0.109 n.s.), CARS “total score” (−16.9% and −20.4%; *F* = 20.429, df 1.469, *p* = 3.0 × 10^−5^; T0 > T1 = T2, *p* < 0.001) and by the CARS subitems “general impression” (−11.6% and −20.4%, *F* = 9.978, 2 df, *p* = 4.3 × 10^−4^; T0 > T1 = T2, *p* < 0.05–0.01), “imitation” (−16.3% and −22.8%, *F* = 7.448, 2 df, *p* = 0.002; T0 = T1 *p* = 0.09 n.s., T0 > T2 *p* = 0.004, T1 = T2 *p* = 0.580 n.s.) and “emotional response” (−20.7% and −22.0%, *F* = 7.420, 2 df, *p* = 0.002; T0 > T1 = T2, *p* < 0.05–0.01), (Figure 2c, Table 2). Changes in all the above‐mentioned behavioral parameters, displayed in Figure 2, were still significant after Bonferroni correction for multiple testing, as detailed in Table 2. Also anxiety showed a prominent decrease but also greater interindividual variability, reaching only nominal significance (CARS item 10 “Fear and anxiety”: −27.7% at T1 and −30.9% at T2; *F* = 4.359, 2 df, *p* = 0.021; T0 > T1 *p* < 0.05, T0 = T2 *p* = 0.119 n.s., T1 = T2 *p* = 0.991 n.s.) (Figure 2d, Table 2).

**TABLE 2:** CARS, CPRS, SRS, and RBS‐R median scores at baseline (T0), and after 1 (T1) and 6 months (T2) of gut mobilization

	T0		T1		T2		Statistics			
	Median	Range	Median	Range	Median	Range	*F* ^b^	*p* Value	*η* ^2^	Power^a^
Childhood autism rating scale (CARS) items										
(1) Social relationship	2.9	2.0–4.0	2.5	1.5–4.0	2.4	1.0–4.0	2.711	0.10	0.145	0.420
(2) Imitation	2.8	1.5–4.0	2.4	1.0–4.0	2.1°°	1.0–4.0	7.448	0.002	0.318	0.919
(3) Emotional response	2.5	1.0–3.5	2.0°	1.0–3.5	1.9°°	1.0–3.0	7.420	0.002	0.317	0.918
(4) Use of body	2.6	1.0–4.0	2.2	1.0–3.5	2.2	1.0–3.0	3.075	0.060	0.161	0.553
(5) Use of objects	2.5	1.0–4.0	2.1°	1.0–3.5	2.2°	1.0–4.0	5.497	0.009	0.256	0.815
(6) Mental & behavioral flexibility	2.3	1.0–3.5	2.0	1.0–3.0	1.9°	1.0–3.0	4.679	0.016	0.226	0.746
(7) Visual response	2.4	1.0–3.5	2.2	1.0–3.0	2.0°	1.0–3.0	4.510	0.019	0.220	0.729
(8) Hearing response	2.1	1.0–3.0	1.9	1.0–3.0	1.7°	1.0–2.5	4.477	0.019	0.219	0.726
(9) Use of senses	2.0	1.0–3.5	1.6	1.0–3.0	1.6°	1.0–3.0	5.195	0.011	0.245	0.792
(10) Fear and anxiety	1.9	1.0–4.0	1.5°	1.0–3.0	1.3	1.0–2.5	4.359	0.021	0.214	0.714
(11) Verbal communication	3.0	1.5–4.0	2.8	1.0–4.0	2.5°	1.5–4.0	3.340	0.048	0.173	0.590
(12) Nonverbal communication	2.6	1.5–4.0	2.0°	1.0–3.5	2.0	1.0–3.5	4.591	0.018	0.223	0.737
(13) Activity level	2.5	1.0–4.0	1.9°°	1.0–3.5	1.8°°	1.0–3.5	9.735	5 × 10^−4^	0.378	0.972
(14) Cognitive level	2.6	1.5–4.0	2.3	1.5–4.0	2.2	1.0–3.5	4.066	0.027	0.203	0.681
(15) General impression	3.0	1.5–4.0	2.7°	1.0–4.0	2.4°°	1.0–4.0	9.978	4.3 × 10^−4^	0.384	0.975
Total score	37.8	27.5–55.0	32.0°°°	19.0–50.0	30.1°°°	17.0–49.0	20.429	3 × 10^−5^	0.561	0.999
Conners' parent rating scale (CPRS) items										
Oppositional	54	41–76	51	41–87	49°	37–72	3.561	0.04	0.182	0.620
Cognitive problems/inattention	62	52–87	62	46–83	60	40–75	2.733	0.08	0.146	0.501
Hyperactivity	66	46–88	60°	42–79	54°°°	38–79	9.515	0.001	0.373	0.969
Anxious‐shy	52	41–83	48	36–81	50	36–71	1.914	0.164	0.107	0.368
Perfectionism	52	37–87	50	37–73	53	37–87	1.625	0.213	0.092	0.318
Social problems	59	42–97	59	41–97	59	44–97	1.934	0.391	0.057	0.203
Psychosomatic	53	42–87	48	40–93	54	42–76	0.211	0.811	0.013	0.080
Conners' ADHD index	69	49–88	65	42–88	59	42–84	2.617	0.089	0.141	0.483
Conners' Global index (restless‐impulsive)	64	47–86	60	50–82	56	44–84	2.196	0.128	0.121	0.415
Conners' Global index (emotional lability)	55	39–82	55	39–88	50	39–75	1.851	0.174	0.104	0.357
Social responsiveness scale (SRS) items										
Social awareness	65.76	45–97	64.95	41–90	56.06°°^	35–72	8.010	0.002	0.334	0.937
Social cognition	78.05	57–104	72.33	53–109	65.94°	45–97	6.028	0.006	0.274	0.851
Social communication	83.33	55–103	79.19	44–115	71.94°°	39–90	7.402	0.002	0.316	0.918
Social motivation	82.10	59–115	76.24	48–115	70.41°	42–96	4.192	0.024	0.208	0.695
Mannerisms	84.71	56–117	75.52°°	53–112	73.53°°	40–112	10.762	2.7 × 10^−4^	0.402	0.983
Total score	86.14	64–109	80.86	54–124	73.76°°°	43–96	11.549	1.7 × 10^−4^	0.419	0.989
Repetitive behavior scale‐revised (RBS‐R) items										
Stereotypied behavior	9.57	1–16	7.52°	1–19	6.00°°	1–16	10.380	3.4 × 10^−4^	0.393	0.980
Self‐injurious behavior	2.43	0–9	1.86	0–9	1.24	0–5	4.999	0.022	0.238	0.663
Compulsive behavior	2.95	0–9	2.10	0–8	1.94	0–7	1.296	0.288	0.075	0.260
Ritualistic/sameness behavior	6.62	0–17	4.95	0–14	4.53°	0–13	5.315	0.010	0.249	0.801
Restricted interests	5.05	0–8	4.29	0–9	3.47°°^	0–8	8.746	0.001	0.353	0.955
Total score	26.62	4–45	20.71	4–39	17.18°°	4–39	11.536	1.7 × 10^−4^	0.419	0.989

*Note*: Median and ranges refer to *N* = 21 at T0 and T1, *N* = 17 at T2. Statistics refer to one‐way ANOVAs for repeated measures on *N* = 17 cases with complete data at all three time points. Nominal *p* values are reported. Significant results after Bonferroni correction for multiple testing are highlighted in gray. Pairwise contrasts following Sidak correction: T0–T1 and T0–T2, °*p* < 0.05, °°*p* < 0.01, °°°*p* < 0.001. T1–T2, ^*p* < 0.05.

^a^ Power with *α* = 0.05.

^b^ *F* (2 d.f.) assuming sfericity based on Mauchly's W statistics, or applying Greenhouse–Geisser or Huynh–Feldt corrections depending on whether *ε* is < or ≥0.75.

**FIGURE 3:**
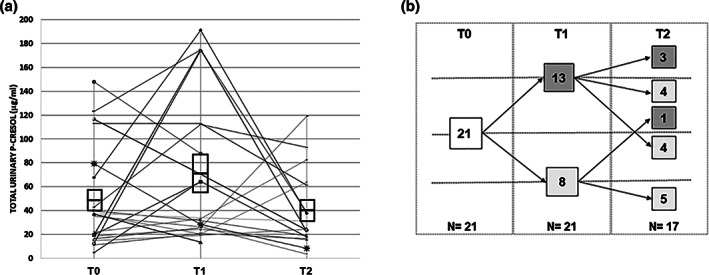
(a) Urinary p‐cresol concentrations at baseline (T0), after 1 (T1), and 6 months (T2) of gut mobilization. Mean ± SEM values are highlighted and boxed, respectively, *N* = 21 at T0–T1 and 17 at T2. (b) Variation in urinary p‐cresol concentrations between baseline (T0), 1 (T1), and 6 months (T2) after gut mobilization. Increasing and decreasing trends compared to the previous time point are highlighted in dark and light gray, respectively

Mean pretreatment urinary p‐cresol concentrations were 48.4 ± 9.3 μg/ml. These levels were not as elevated as previously found in similar samples of young autistic children, especially if chronically constipated, instead they were comparable to control levels reported in previous studies (70.3 ± 6.7 μg/ml and 52.0 ± 7.8 μg/ml in Altieri et al., 2011, and in Gabriele et al., 2014, respectively). During the course of gut mobilization, mean urinary p‐cresol displayed highly variable trajectories (Figure 3a), with an overall non‐significant mean increase to 71.7 ± 13.3 at T1 and decrease to 39.9 ± 7.9 at T2 (one‐way ANOVA for repeated measures: *F* = 2.214, 2 d.f., *p* = 0.126; *η* ^2^ = 0.12, 1 − *β* = 0.418). Trend‐wise, higher p‐cresol concentrations were recorded at T1 compared to T0 in 13/21 (61.9%) children, whereas p‐cresol decreased at T2 over T1 in 13/17 (76.5%) children (Figure 3b). At the end of the 6‐month follow‐up, T2 urinary p‐cresol concentrations were below baseline T0 levels in 9/17 (52.3%) children (Figure 3b). Only 3/17 (17.6%) children displayed progressively increasing p‐cresol concentrations at T0–T1–T2 (Figure 3b). Interestingly, median levels of urinary p‐cresol decreased over the time (36.5, 34.3, and 24.6 ng/ml at T0, T1, and T2, respectively), confirming the presence of several outliers at T1 reaching the highest concentrations recorded in this study (up to 191.0 ng/ml, more than 13 folds over baseline values) (Figure S1). Five subjects receiving antibiotic therapy for disorders not related to the GI tract (i.e., upper airway infections), displayed some of the most profound decreases in urinary p‐cresol in three children and a slight increase in two children.

Urinary p‐cresol was not directly correlated with Bristol stool scale scores, nor with any behavioral measure, both in terms of absolute values and of variation between time points (“delta” T1 − T0, T2 − T1, and T2 − T0). Regression analyses were also essentially negative. Only T1 − T0 differences in CPRS anxiety scores were nominally correlated with T1 − T0 differences in urinary p‐cresol concentrations and in Bristol stool scale scores (both *p* < 0.05) (Table 3). Instead, T2 − T0 differences in CPRS ADHD index and CARS imitation scores were nominally correlated with T2 − T0 differences in Bristol stool scale scores (*p* < 0.01) (Table 3). These results do not support major contributions by p‐cresol and its derivatives in producing the sizable behavioral improvement observed following gut mobilization in ASD children.

**TABLE 3:** Linear regression analyses

	Delta T1–T0	Delta T2–T1	Delta T2–T0
CARS imitation			
Total	0.411	0.203	0.025*
P‐cresol	0.391	0.617	0.215
Bristol stool scale	0.291	0.079	0.013*
CPRS anxiety			
Total	0.015*	0.141	0.149
P‐cresol	0.044*	0.782	0.056
Bristol stool scale	0.031*	0.053	0.901
CPRS ADHD‐index			
Total	0.233	0.070	0.030*
P‐cresol	0.094	0.066	0.962
Bristol stool scale	0.776	0.066	0.009**

^*^ *p* < 0.05.

^**^ *p* < 0.01.

## DISCUSSION

This study was designed with the primary purpose of measuring the behavioral consequences of gut mobilization in chronically constipated autistic children. Secondly, we assessed the possible co‐variation of gut‐derived total p‐cresol with the severity of core ASD symptoms and of frequently co‐morbid hyperactivity and anxiety. To this aim, behavioral patterns, stool consistency, and urinary p‐cresol were measured and correlated in chronically constipated ASD children before and after intestinal mobilization. As expected, bowel transit was reliably accelerated by the consumption of PEG (Figure 1), a long linear polymer of ethylene glycol monomers (high‐molecular weight polyethylene glycol) that binds water molecules through hydrogen bonds. Gut mobilization was consistently followed by a progressive and sizable decrease in hyperactivity, anxiety, social interaction deficits, and stereotypic behaviors over six months (Figure 2a–d). This behavioral improvement was thus not only statistically significant, but also clinically meaningful. In fact, on the one hand, ADHD and anxiety disorders are among the most frequent co‐morbidities in autistic patients, diagnosed in 33%–37% and in 39.6% of ASD cases, respectively (Berenguer‐Forner et al., 2015; Van Steensel et al., 2011), while social interaction deficits and repetitive movements represent two of the hallmarks of an ASD diagnosis. On the other hand, by comparison, the 7.7 point mean decrease in CARS total score recorded here after 6 months of gut mobilization (Figure 2c, Table 2) is larger than the mean decrease of approximately five points observed following long‐term treatment with approved pharmacological agents like risperidone (Maneeton et al., 2018; Persico et al., 2021) or with promising experimental drugs, like bumetanide (Lemonnier et al., 2012). The lack of blinding in this study has probably contributed to enhance the magnitude of clinical improvement, yet gut mobilization had a clear positive effect on multiple aspects of children's behavior. These results also match the behavioral response to gut mobilization we usually observe in clinical practice, as well as convergent observations by parents and therapists. This improvement, though partial, often times allows a dosage reduction of ongoing psychopharmacological treatments or prevents their prescription altogether.

Meanwhile, trends in urinary p‐cresol excretion displayed large intra‐ and inter‐individual variability, with a predominant increase at one month and decrease at six months. The most plausible explanation for these changes is the effect of PEG treatment on the microbiota. PEG, when administered orally, increases the volume of gut content, yielding faster bowel transit in both the small intestine and the colon, thus exerting laxative effects. It is not fermented by the intestinal microbiota but, like other interventions aimed to accelerate intestinal transit time, it determines alterations in the overall microbial community composition of the distal gut (Kashyap et al., 2013). In general, PEG consumption can affect microbiota composition through different mechanisms, with diverse and putatively opposite effects. On the one hand, a faster intestinal transit can be predicted to reduce the extent of protein fermentation, blunting urinary p‐cresol levels. Indeed, the fermentation of complex polysaccharides occurs in the first sections of the colon (i.e., ascending and transversal colon), whereas, when carbohydrates are depleted, proteins are mostly fermented in the descending part of the colon (MacFarlane et al., 1992). Stagnation of intestinal content in the colon fosters the exhaustion of saccharolytic metabolism and prompts a putrefactive metabolism, resulting in greater p‐cresol production. On the other hand, a shorter transit time affects microbial composition of the gut content without altering the flux of nutrients, and this unpredictable change can favor taxa able to produce p‐cresol (Kashyap et al., 2013). Our results indicate that the former mechanism seemingly tends to prevail, but responses are nonetheless highly personalized.

The highly variable trajectories recorded in urinary p‐cresol concentrations, contrast with the consistent decrease recorded in behavioral symptoms, resulting in marginal or no correlation between biochemical and behavioral parameters. Hence, variation in p‐cresol absorption may not play a major role in modulating behavioral changes following gut mobilization in ASD. The limited effects of a single gut‐derived compound on the overall behavioral expression of a complex human neurodevelopmental disorder like ASD are not entirely surprising. Perhaps more surprising are the 8–12 pt mean decreases in SRS, CARS, RBS, and CRPS scale scores produced by a relatively simple intervention like gut mobilization in chronically constipated autistic children (Figure 2a–d). In previous studies, the presence of gastrointestinal disorders, especially constipation, has been consistently associated with more severe behavioral symptoms, ranging in different cohorts from rigid–compulsive behaviors, to motor stereotypies, social impairment, expressive language deficits, self‐injurious behaviors, sensory over‐responsivity, somatic complaints, anxiety, reduced sleep duration and quality (Chakraborty et al., 2021; Ferguson et al., 2019; Fulceri et al., 2016; Gorrindo et al., 2012; Mazurek et al., 2013; Peters et al., 2014; Prosperi et al., 2019; Restrepo et al., 2020). Three pilot studies have provided preliminary evidence of improvement in irritability and/or in several behavioral parameters following gut mobilization obtained using prebiotics and/or probiotics (Grimaldi et al., 2018; Inoue et al., 2019; Sanctuary et al., 2019). Sustained and progressive reductions in autism severity and increased adaptive behaviors were recorded in an open trial of 18 autistic children followed‐up for two years after undergoing microbiota transfer therapy (Kang et al., 2017, 2019). The present results thus provide strong additional evidence that gut mobilization, obtained applying a PEG protocol of widespread use in clinical settings, yields broad‐based improvements in multiple behavioral domains of chronically constipated autistic children.

The present study has several limitations. Its open design reflects closely what happens in clinical practice, but likely enhances behavioral effect sizes due to lack of blinding. Indeed, both psychologists administering the CARS and parents filling in the other questionnaires were not blind to active treatment status, which is difficult to conceal when promoting gut mobilization. Moreover, we cannot exclude that some contribution to behavioral improvement may be provided by generic relief from gut discomfort and not necessarily by the reduced absorption of specific neuroactive compounds produced by the gut microbiota. Our study was not designed to address this question, rather to assess the behavioral consequences of gut mobilization and their degree of correlation with urinary p‐cresol. We deem unlikely that non‐specific amelioration of bowel discomfort represents the major source of behavioral improvement. In fact, pain due to colonic contractions is typically crampiform, and aching due to anal fissures is associated with bowel emptying. In either case, pain is typically phasic and would be predicted to translate into bouts of hyperactivity and self‐aggressive behavior, as frequently observed, for example, in clinical settings attended by non‐verbal, severely autistic individuals with periodic constipation. Instead, the behavioral pattern we observed in these children and improved here by gut mobilization is characterized by a continuous ADHD‐like hyperactivity, by chronic anxiety and stable autistic behaviors. Nonetheless, a more complex design, including a control sample of typically developing (TD) children with chronic constipation, careful monitoring of subjective abdominal pain and parallel urinary and/or foecal metabolomics of ASD and TD children with chronic constipation undergoing gut mobilization, will be needed to substantiate these clinical observations and to estimate the relative weight of specific versus non‐specific factors in determining the observed behavioral outcome.

Secondly, our patient sample surprisingly displays normal pre‐treatment urinary p‐cresol concentrations, consistent with control levels from our previous studies (Altieri et al., 2011; Gabriele et al., 2014). Consequently, our sample size, which was based on prior studies, is clearly underpowered in detecting significant changes in p‐cresol, which would have required here as many as 70 cases assessed at all three time points to reach a statistical power of 0.8. Conceivably, this difference could be due to several factors which were not controlled in our study design, like intra‐ and inter‐individual variability of p‐cresol precursors in the diet. Protein‐rich diets, particularly those containing red meat, favor excessive production of microbial products derived from protein fermentation, including p‐cresol, mainly derived from catabolism of the aromatic amino acids phenylalanine and tyrosine. In general, the modification of dietary patterns, such as including dietary carbohydrates, shows an inverse relationship with the concentration of this putrefactive fecal compound (Russell et al., 2011). It is important to consider that every factor that affects microbiota composition can influence the populations of both microorganisms that produce and degrade p‐cresol, and can impact directly or indirectly on the availability of the substrates tyrosine and phenylalanine. For instance, fasting in rats enhances the production of p‐cresol as a consequence of the increased concentration of endogenous protein in the caecum (Kawakami et al., 2007). As a whole, shifts in individual microbial composition can modify the functional repertoire of gut microbiota, affecting important host interactions that rely on microbial metabolites, and leading to the overproduction or consumption of systemic metabolites, with opposite trends in terms of concentrations.

Thirdly, total urinary p‐cresol, as measured in the present study, is almost entirely made up of p‐cresylsulfate, with p‐cresylglucuronide and unconjugated free p‐cresol together reaching at most 5% on average. P‐cresylsulfate actually represents the main uremic toxin responsible for negatively affecting the CNS in chronic renal disease (Persico & Napolioni, 2013). We have previously shown that the increases in urinary p‐cresol concentrations recorded in ASD involve all three compounds (Gabriele et al., 2014). The biotransformation of p‐cresol into p‐cresylsulfate mainly occurs in the colonic epithelium, and to a lesser extent in the liver. Therefore, though unlikely, we cannot exclude that gut mobilization may influence p‐cresol conjugation rates and modify the relative abundance of the three metabolites, which have not been separately measured in the present study.

Finally, our results support minor contributions by p‐cresol to the changes in behavioral symptoms observed following the specific paradigm of gut mobilization. However, on the one hand this conclusion cannot be generalized as to exclude possible acute or chronic “steady state” p‐cresol effects on autistic behaviors, as supported by rodent models (Bermudez‐Martin et al., 2021; Pascucci et al., 2020). On the other hand, p‐cresol is definitely not the sole neuroactive exogenous compound produced by gut bacteria able to negatively influence animal and human behavior. Urinary metabolomic studies have consistently unveiled an excess of compounds synthesized by the gut microbiota potentially able to exert behavioral effects, like indolyl 3‐acetic acid and indolyl lactate (Gevi et al., 2016; Mussap et al., 2020). Another compound linked to a variety of behavioral, immune, and mitochondrial effects in rodent models of ASD is propionic acid, a short chain fatty acid produced by anaerobic gut bacteria including clostridia and propionibacteria (MacFabe, 2015). Elevated urinary and foecal levels of propionic acid in autistic children, as compared to neurotypical controls, have been detected in some, though not all studies (De Angelis et al., 2013; Kang et al., 2018). Nonetheless, the consequences of gut mobilization in constipated autistic children clearly merit exploration using an unbiased urinary and/or foecal metabolomic approach, to capture the full breath of biochemical changes and of gut‐derived factors influencing behavior and contributing to symptom severity.

Despite these limitations, our study documents the behavioral benefits of gut mobilization in chronically constipated young autistic children using several converging psychometric tools (Figure 2a–d, Table 2). The correction of chronic constipation, whenever diagnosed in an autistic child, represents a simple, effective, and reasonable approach to at least partially improve core and co‐morbid behavioral symptoms. Defining the relative contributions of reduced abdominal discomfort (Buie et al., 2010) and of changes in microbiota composition produced by gut mobilization will require parallel longitudinal assessments of metabolome and microbiota in larger samples of chronically constipated autistic children. Contrary to animal models of ASD (Bermudez‐Martin et al., 2021; Pascucci et al., 2020), p‐cresol appears only marginally related to behavioral symptoms in the present study, but an ensemble of gut‐derived neurotoxic compounds produced by a skewed microbiota may well exert in humans a sizable effect. In this case, favorable modifications of the gut microbiota through intervention protocols, ranging from microbiota transfer therapy to more clinically manageable prebiotics, could potentially benefit also autistic children even in the absence of overt stool or gastrointestinal issues.

## CONFLICT OF INTEREST

The authors declare that they have no conflict of interest.

## ETHICS STATEMENT

This study conforms to the standards of the Declaration of Helsinki and is part of a large study protocol regarding the search for diagnostic/prognostic biomarkers of Autism Spectrum Disorder, reviewed and approved by the Ethics Committee of the University of Messina and of the “G. Martino” University Hospital (Messina, Italy). Written informed consent was obtained from both parents of all the children who participated in this study.

## Supporting information


**FIGURE S1**: Boxplot of urinary p‐cresol concentration ratios (T1/T0, T2/T1, T2/T0). Outliers at T1 display the highest p‐cresol levels recorded in this sample (see text).
