# Supplementary figures and images for: Gut mobilization improves behavioral symptoms and modulates urinary p‐cresol in chronically constipated autistic children: A prospective study
Source: Autism Res. 2021 Nov 23;15(1):56–69. doi: 10.1002/aur.2639 (PMC9299106; doi:10.1002/aur.2639)

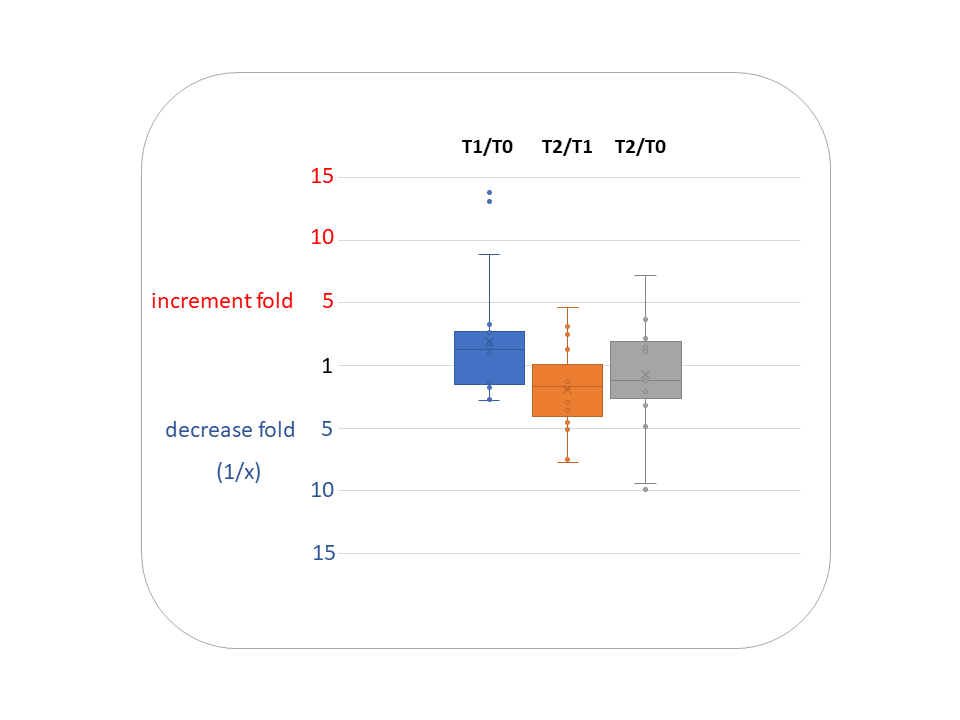

Supplement: Supplementary file 1 — FIGURE S1: Boxplot of urinary p‐cresol concentration ratios (T1/T0, T2/T1, T2/T0). Outliers at T1 display the highest p‐cresol levels recorded in this sample (see text). [file AUR-15-56-s001.tif]
